# IncHI2 Plasmids Are Predominant in Antibiotic-Resistant *Salmonella* Isolates

**DOI:** 10.3389/fmicb.2016.01566

**Published:** 2016-09-30

**Authors:** Wenyao Chen, Tingzi Fang, Xiujuan Zhou, Daofeng Zhang, Xianming Shi, Chunlei Shi

**Affiliations:** Ministry of Science and Technology–United States Department of Agriculture Joint Research Center for Food Safety, School of Agriculture and Biology, Shanghai Jiao Tong UniversityShanghai, China

**Keywords:** *Salmonella*, antibiotic resistance, replicon typing, plasmid curing, IncHI2, plasmid sequencing

## Abstract

The wide usage of antibiotics contributes to the increase in the prevalence of antibiotic-resistant *Salmonella*. Plasmids play a critical role in horizontal transfer of antibiotic resistance markers in *Salmonella*. This study aimed to screen and characterize plasmid profiles responsible for antibiotic resistance in *Salmonella* and ultimately to clarify the molecular mechanism of transferable plasmid-mediated antibiotic resistance. A total of 226 *Salmonella* isolates were examined for antimicrobial susceptibility by a disk diffusion method. Thirty-two isolates (14.2%) were resistant to at least one antibiotic. The presence of plasmid-mediated quinolone resistance (PMQR) genes and *β*-lactamase genes were established by PCR amplification. PCR-based replicon typing revealed that these 32 isolates represented seven plasmid incompatibility groups (IncP, HI2, A/C, FIIs, FIA, FIB, and I1), and the IncHI2 (59.4%) was predominant. Antibiotic resistance markers located on plasmids were identified through plasmid curing. Fifteen phenotypic variants were obtained with the curing efficiency of 46.9% (15/32). The cured plasmids mainly belong to the HI2 incompatibility group. The elimination of IncHI2 plasmids correlated with the loss of *β*-lactamase genes (*bla*_OXA-1_ and *bla*_TEM-1_) and PMQR genes (*qnrA* and *aac(6′)-Ib-cr*). Both IncHI2 and IncI1 plasmids in a *S. enterica* serovar Indiana isolate SJTUF 10584 were lost by curing. The *bla*_CMY -2_-carrying plasmid pS10584 from SJTUF 10584 was fully sequenced. Sequence analysis revealed that it possessed a plasmid scaffold typical for IncI1 plasmids with the unique genetic arrangement of IS*1294*-ΔIS*Ecp1*-*bla*_CMY -2_-*blc*-*sugE*-Δ*ecnR* inserted into the colicin gene *cia*. These data suggested that IncHI2 was the major plasmid lineage contributing to the dissemination of antibiotic resistance in *Salmonella* and the activity of multiple mobile genetic elements may contribute to antibiotic resistance evolution and dissemination between different plasmid replicons.

## Introduction

*Salmonella* is recognized as the predominant pathogen implicated in bacterial foodborne diseases worldwide (Yang et al., 2016). The emergence of multidrug-resistant (MDR) *Salmonella* strains due to the prolonged and extensive usage of antibiotics has become a public health issue. Such resistance toward medically significant antimicrobial agents including fluoroquinolones and extended-spectrum cephalosporins (ESCs) that are regarded as primary treatment options for bacterial infections will make it difficult for antibiotic therapy due to the reduced efficiency of empirical strategies as well as limited choice of treatment (Wong et al., 2014; Folster et al., 2015).

The dissemination of undesirable antibiotic resistance in Gram-negative bacteria has been largely attributed to the acquisition of multiple plasmid-located antibiotic resistance genes by horizontal gene transfer (HGT; Carattoli, 2013; Wang et al., 2015). The emergence of plasmid-mediated quinolone resistance (PMQR) predominantly encoded by *qnr* variants, *aac(6′)-Ib-cr*, *qepA*, and *oqxAB* genes in *Salmonella*, has further facilitated selection of high-level chromosomal quinolone resistance *via* mutations in topoisomerase genes, as well as the wide spread of quinolone resistance *via* HGT (Jiang et al., 2014; Skov et al., 2015). In addition, the apparent correlation between the PMQR genes (such as *qnr* or *aac(6′)-Ib-cr* genes) and extended-spectrum *β*-lactamase (ESBL) genes or *ampC* genes on the same plasmid and their association with other antibiotic resistance genes have raised particular global concern, since their transmission could be driven by other mobile genetic elements located on plasmids among various bacterial species (Wang et al., 2013; Jiang et al., 2014). Particular plasmid families associate with the emergence and dissemination of specific antibiotic resistance traits with differential prevalence and distribution (Johnson et al., 2007; Carattoli, 2013). Thus, characterization of plasmids based on PCR-based replicon typing (PBRT) is an indispensable part of plasmid epidemiological surveillance enhancing discrimination between *Salmonella* strains as well as tracing the spread and evolution of antibiotic resistance genes (Wang et al., 2013). Certain replicon types were found to be associated with MDR as well as with bacterial disease outbreaks (Huang et al., 2012). It has been reported that plasmids with IncA/C, B/O, HI1, HI2, I1, N, F, and P replicons are often associated with MDR in *Salmonella*, while many of them are found to be co-resident in some MDR *Salmonella* strains (Poole et al., 2009; Glenn et al., 2013).

Transmissible plasmids contain a full set of conjugation-encoding genes facilitating their spread over large taxonomic distances, and they harbor drug-resistance determinants, virulence factors and addiction systems promoting their stability and maintenance in bacterial hosts under different environmental conditions. It is essential to clarify the roles of these genetic elements and the corresponding surrounding genetic structures by plasmid sequencing in order to trace the transmission and persistence of antibiotic resistance (Carattoli, 2013; Wang et al., 2015).

The aim of this study was to screen and characterize plasmid replicon profiles containing PMQR and *β*-lactamase genes in *Salmonella* so as to clarify molecular mechanism of transferable plasmid-mediated antibiotic resistance.

## Materials and Methods

### *Salmonella* Isolates

A total of 226 *Salmonella* isolates were used in this study, and all the non-susceptible strains are listed in Supplementary Table S1, of which 39 were food isolates and 39 were clinical isolates. Among these *Salmonella* isolates, clinical isolates were collected by Shanghai Municipal Center for Disease Control and Prevention and Wuhan Municipal Center for Disease Control and Prevention, while food isolates were collected from beef, poultry, pork, shrimp, vegetables, fresh juice, and shellfish. PCR-based serotyping was performed as previously described (Ranieri et al., 2013). Two different primer pairs and an additional primer pair were used in this study (Supplementary Table S2). The detailed information of these isolates is listed in Supplementary Table S1.

### Antimicrobial Susceptibility Testing

All 226 *Salmonella* isolates underwent antimicrobial susceptibility testing using the disk diffusion method against a panel of 18 antibiotics, according to the standards and guidelines recommended by the Clinical and Laboratory Standards Institute (CLSI; CLSI, 2013). A total of 18 antibiotic disks (Oxoid Ltd., Basingstoke, UK) that included ampicillin (AMP, 10 μg), piperacillin/tazobactam (TZP, 100/10 μg), ampicillin/sulbactam (SAM, 10/10 μg), ceftriaxone (CRO, 30 μg), ceftazidime (CAZ, 30 μg), cefepime (FEP, 30 μg), cefotetan (CTT, 30 μg), aztreonam (ATM, 30 μg), cephazolin (CZO, 30 μg), ciprofloxacin (CIP, 5 μg), imipenem (IPM, 10 μg), amikacin (AMK, 30 μg), gentamicin (GEN, 10 μg), tobramycin (TOB, 10 μg), ertapenem (ETP, 10 μg), levofloxacin (LEV, 5 μg), nitrofurantoin (NIT, 300 μg), and sulfamethoxazole/trimethoprim (SXT, 23.75/1.25 μg), were assessed. *Escherichia coli* ATCC 25922 was used as the control strain. Isolates were defined as MDR if they were resistant to at least three different classes of antibiotics.

### Detection of PMQR and β-Lactamase Genes

Genomic DNA of *Salmonella* isolates was purified by the cetyltrimethylammonium bromide (CTAB) method as described elsewhere (Wilson, 2001). The antibiotic-resistant isolates identified by antimicrobial susceptibility testing were screened for the presence of PMQR determinants (*qnrA, qnrB, qnrS, qnrC, qnrD, aac (6′)-Ib-cr*) and *β*-lactamase genes (*bla*_TEM-1_, *bla*_OXA-1_, *bla*_PSE-1_, and *bla*_CMY -2_) by simplex PCR amplification. Primers targeting above antibiotic resistance genes were listed in Supplementary Table S3.

### Plasmid Replicon Typing

Plasmid incompatibility (Inc) groups were assigned by PBRT using genomic DNA of *Salmonella* isolates as template. Amplification by PCR was performed with eighteen specific primer pairs designed for FIA, FIB, FIC, HI1, HI2, I1, L/M, N, P, W, T, A/C, K, B/O, X, Y, F, and FIIA basic replicons, and the corresponding protocol was previously described (Carattoli et al., 2005). Resulting amplicons were sequenced by Shanghai Majorbio Bio-pharm Technology Co., Ltd. Comparative analysis of nucleotide sequences was performed using the BLAST program at the National Center for Biotechnology Information (NCBI) site^1^.

### Plasmid Curing

To determine if the antibiotic resistance observed in this study was plasmid-mediated, the identified antibiotic-resistant *Salmonella* isolates were subjected to a curing treatment using sodium dodecyl sulphate (SDS), according to Tomoeda et al. (1968) with modifications. Briefly, the overnight culture in Luria–Bertani (LB) broth was inoculated (1:10^4^ dilution) in LB broth containing 5% SDS and incubated for 48 h at 44.5°C with constant agitation. Aliquots of the cultures were then appropriately diluted and spread on LB agar plates without antibiotics and incubated at 37°C for 16–18 h. The resulting single clones were streaked onto two LB agar plates with or without ampicillin. Because ampicillin was the most prevalent among antibiotic-resistant isolates, for the screening convenience of those plasmid-loss mutants, ampicillin was used as the priority selective marker. The LB agar plates were incubated at 37°C overnight, and ampicillin-susceptible clones were considered positive for loss of the incompatible plasmid. After treatment with the curing agent, the putative cured derivatives were examined for the antibiotic susceptibility profile using the same set of antibiotics, and for the presence of related antibiotic resistance genes as well as plasmid replicon type by PCR method as described above.

### Plasmid Sequencing and Analysis

The *Salmonella* isolate SJTUF 10584 identified in this study was chosen as the plasmid donor for its complete loss of plasmids and MDR after one single trial of plasmid curing. The plasmid was obtained through conjugation experiments by the liquid mating assay using rifampin-resistant *E. coli* Nk5449 as the recipient strain as previously described (Zurfluh et al., 2014). Transconjugants were selected on LB agar plates containing ampicillin (100 μg/ml) and rifampin (200 μg/ml). Antimicrobial susceptibility tests and antibiotic resistance gene detection were performed to confirm the plasmid transfer, followed by PBRT to test which plasmid and antibiotic resistance markers were transferred.

Plasmid DNA was purified from the transconjugant by the Qiagen Plasmid Midi Kit (Qiagen, Germany) according to the manufacturer’s protocol. Plasmid sequencing was performed on the IIIumina Hiseq sequencing platform at the Shanghai Biotechnology Corporation (Shanghai, China). The complete sequence was annotated using the NCBI Prokaryotic Genome Annotation Pipeline (PGAP), followed by manual inspection. Each predicted protein was further confirmed against the NCBI all-protein database using BLASTP^1^. Insertion sequences and repetitive elements were identified using IS finder^2^. DNA sequence comparisons and alignments were performed using BLAST^1^ and Geneious software (version 9.1.2; Kearse et al., 2012), and a schematic plasmid map was constructed with WinPlas 2.7 software.

### Nucleotide Sequence Accession Number

The complete sequence of the plasmid pS10584 has been deposited in Genbank/EMBL/DDBJ with accession number KX058576.

## Results and Discussion

### Antimicrobial Susceptibility

**FIGURE 1:**
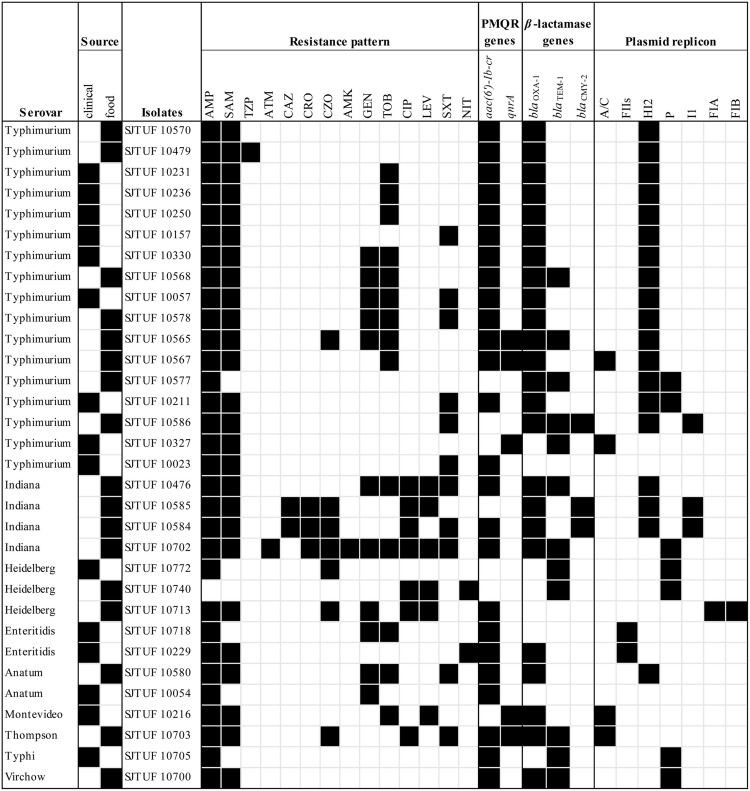
**A heat-map summary of the serovars, the sources, the antibiotic resistance phenotypes and genotypes, and the corresponding Inc plasmid type(s) for 32 drug-resistant *Salmonella* isolates.** Black and white squares denotes the presence and absence of a particular feature, respectively. The antibiotics listed are abbreviated as follows: AMP, ampicillin; TZP, piperacillin/tazobactam; SAM, ampicillin/sulbactam; CRO, ceftriaxone; CAZ, ceftazidime; ATM, aztreonam; CZO, cephazolin; CIP, ciprofloxacin; AMK, amikacin; GEN, gentamicin; TOB, tobramycin; LEV, levofloxacin; NIT, nitrofurantoin; SXT, sulfamethoxazole/trimethoprim.

Among 226 *Salmonella* isolates, 14.2% (32/226) demonstrated resistance to at least one antibiotic and 11.1% (25/226) were resistant to ≥3 antibiotics. In addition, 4.9% (11/226) showed MDR phenotypes. It’s noteworthy that the strain SJTUF 10702 isolated from chicken exhibited strong resistance to 11 antibiotics. In terms of 32 antibiotic-resistant isolates (**Figure 1**), 25.0% (8/32) presented the quinolone resistance phenotype (LEV or CIP) while 96.9% (31/32) were resistant to the five tested *β*-lactams (AMP, SAM, CZO, CRO, and CAZ) alone or in combination. Moreover, resistance to individual agents was most frequently observed to AMP (31/32, 96.9%) and SAM (26/32, 81.3%), followed by TOB (14/32, 43.8%) and SXT (11/32, 34.4%). No resistance was detected to CTT, FEP, IPM, and ETP.

### Prevalence of PMQR and β-Lactamase Genes

Amongst the 32 antibiotic-resistant *Salmonella* isolates, the PMQR genes *aac(6′)-Ib-cr* and *qnrA* were present alone or in combination in 25 (78.1%) and 5 (15.6%) isolates, respectively; three isolates harbored both two determinants (**Figure 1**). The *qnrB*, *qnrS*, *qnrC*, and *qnrD* genes were not detected in any of the tested isolates. In terms of *β*-lactamase genes detected, 75.0% (24/32) were positive for *bla*_OXA-1_, 37.5% (12/32) for *bla*_TEM-1_ and 9.4% (3/32) for *bla*_CMY -2_. In addition, seven isolates simultaneously carried *bla*_OXA-1_ and *bla*_TEM-1_, and two carried *bla*_OXA-1_ and *bla*_CMY -2_, while one isolate harbored all these three determinants. No isolates contained *bla*_PSE-1_. Of these 27 PMQR-bearing isolates, 23 isolates (85.2%) also carried at least one *β*-lactamase gene with the prevalent pattern of *aac(6′)-Ib-cr-bla*_OXA-1_ (12/23, 52.2%) and *aac(6′)-Ib-cr-bla*_OXA-1_-*bla*_TEM-1_ (4/23, 17.4%).

### Plasmid Replicon Typing

Plasmid replicons detected among 32 antibiotic-resistant *Salmonella* isolates are listed in **Figure 1**. Overall, seven different replicons of the 18 classic replicon types associated with *Enterobacteriaceae* were identified in all the isolates except SJTUF 10023 and SJTUF 10054. The two untypeable isolates may possess divergent or novel replicons (Carattoli, 2009). PBRT demonstrated that IncHI2 (19/32, 59.4%) was the predominant type while the other Inc groups such as P (7/32, 21.9%), A/C (4/32, 12.5%), I1 (3/32, 9.4%), FII_S_ (2/32, 6.25%), FIA (1/32, 3.1%), and FIB (1/32, 3.1%) were just present in few isolates. Seven isolates were mixed of two replicon types, including the combinations of HI2 and I1 (3/7, 42.9%), HI2 and P (2/7, 28.6%), HI2 and A/C (1/7, 14.3%), and FIA and FIB (1/7, 14.3%). Inc groups HI2, I1, A/C, P, FIA, and FIB were found to be associated with MDR isolates, in agreement with previous studies (Poole et al., 2009; Glenn et al., 2013). In addition, IncHI2 was the dominant replicon type both among MDR isolates (7/11, 63.6%) and *Salmonella* Typhimurium isolates (15/17, 88.24%). When comparing multiple antibiotic resistance genes with replicon types, 86.7% (19/22) of *bla*_OXA-1_ positive isolates as well as 81.8% (9/11) of *bla*_OXA-1_-*aac(6′)-Ib-cr* positive isolates were carrying IncHI2 plasmids. And all the three *bla*_CMY -2_ positive isolates contained IncHI2 and IncI1 plasmids simultaneously. In addition, *bla*_TEM-1_ positive isolates tended to carry the IncHI2 (5/12, 41.7%) and IncP (6/12, 50.0%) plasmids while *qnrA* positive isolates were likely apt to carry IncA/C plasmids (4/5, 80.0%).

### Plasmid Curing and Antibiotic Resistance Localization

**FIGURE 2:**
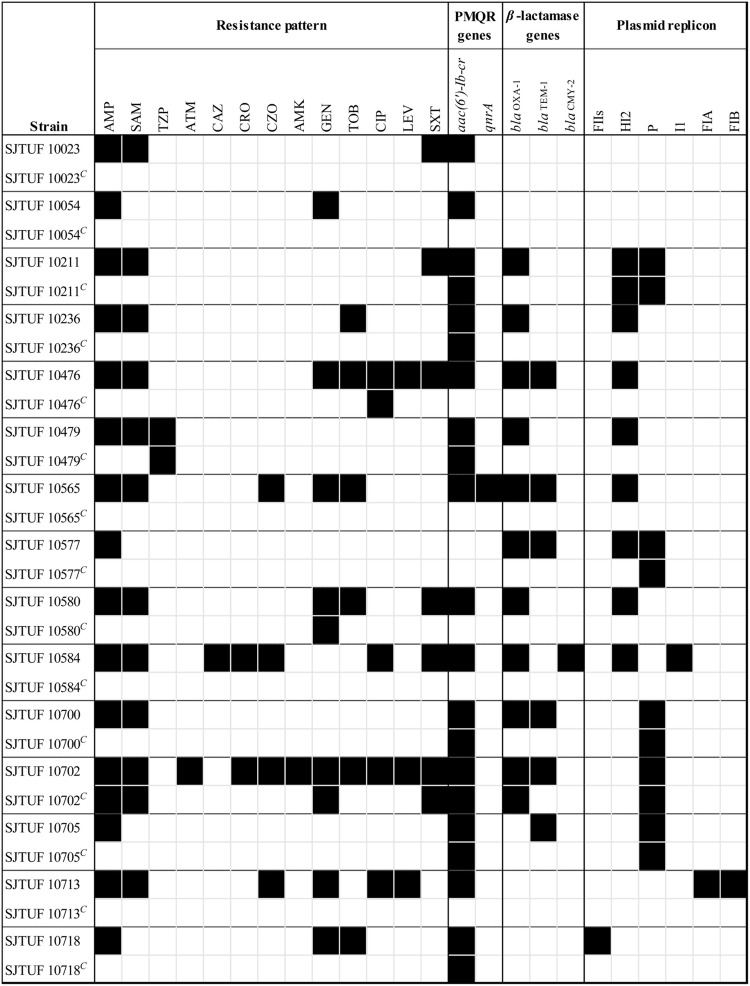
**A heat-map showing the changes of *Salmonella* isolates after plasmid curing based on antibiotic resistance profiles as well as plasmid replicon type(s).** “*C*” denoted the cured variant. Black and white squares denotes the presence and absence of a particular feature, respectively. The antibiotics listed are abbreviated as follows: AMP, ampicillin; TZP, piperacillin/tazobactam; SAM, ampicillin/sulbactam; CRO, ceftriaxone; CAZ, ceftazidime; ATM, aztreonam; CZO, cephazolin; CIP, ciprofloxacin; AMK, amikacin; GEN, gentamicin; TOB, tobramycin; LEV, levofloxacin; SXT, sulfamethoxazole/trimethoprim.

All the 32 antibiotic-resistant *Salmonella* isolates underwent plasmid curing using 5% SDS as well as elevated temperature (44.5°C) for 48 h to ensure that the antibiotic resistance profile was related to the plasmid. Fifteen phenotypic variants were obtained after up to 10 trials of curing (**Figure 2**), and the curing efficiency was 46.9% (15/32). The cured plasmids mainly belonged to IncHI2 group (7/8, 87.5%), followed by IncF group (3/3, 100%), and IncI1 group (1/1, 100%); however, no strain showed curing of IncP group plasmids (0/5, 0%). When attributing the loss of resistance phenotypes to the loss of plasmids, we could infer that the antibiotic resistant phenotypes AMP-SAM-TOB of SJTUF 10236, AMP-SAM-GEN-TOB-LEV-SXT of SJTUF 10476, AMP-SAM of SJTUF 10479, AMP-SAM-GEN-TOB-CZO of SJTUF 10565, AMP of SJTUF 10577, and AMP-SAM-TOB-SXT of SJTUF 10580 were all related to IncHI2 plasmids while AMP-SAM-CZO-CAZ-CRO-CIP-SXT of SJTUF 10584 was determined together by IncI1 and IncHI2 plasmids. And the resistant phenotype AMP-SAM-CZO-GEN-CIP-LEV of SJTUF 10713 and AMP-GEN-TOB of SJTUF 10718 were both determined by IncF plasmids. In addition, the elimination of IncHI2 plasmids correlated with the loss of *β*-lactamase genes (*bla*_OXA-1_ and *bla*_TEM-1_) as well as PMQR genes (*qnrA* and *aac(6′)-Ib-cr*), and the loss of IncFIA and FIB plasmids resulted in the absence of the *aac(6′)-Ib-cr* gene. Furthermore, four cured variants (SJTUF 10211*^C^*, 10700*^C^*, 10702*^C^* and 10705*^C^*) lost antibiotic resistance genes as well as drug resistance phenotypes but still retained the identified plasmids (**Figure 2**). This may be due to the loss of divergent or novel replicons which were untypeable by the current PBRT scheme. The cured SJTUF 10577*^C^* lost the AMP resistance phenotype, *bla* genes, and the IncHI2 plasmid simultaneously, even though it retained the IncP plasmid. The function of IncP plasmids in antibiotic resistance needs further study.

Particular plasmids belonging to the Inc families usually carry multiple physically linked genetic determinants, playing a major role in the diffusion of specific antibiotic resistance genes among different bacteria (Carattoli, 2013; Wang et al., 2013). In this study, we characterized IncHI2 plasmids as the main vehicle in horizontal transfer of *β*-lactamase genes (*bla*_OXA-1_ and *bla*_TEM-1_) as well as PMQR genes (*qnrA* and *acc(6′)-ib-cr*). The co-existence of *aac(6′)-Ib-cr* with *bla*_OXA-1_, with or without *bla*_TEM-1_ was also identified. IncHI2 plasmids are known to play a significant role in the acquisition of antibiotic resistance, and they have recently been implicated in the dissemination of genes encoding extended-spectrum *β*-lactamase (Cain and Hall, 2012). In contrast, IncA/C plasmids are prevalent in several MDR *Salmonella* serovars and closely linked to the expansion of MDR in the United States (Folster et al., 2010, 2015). We also discovered the high prevalence of IncHI2 plasmids in *S.* Typhimurium, similar to the report from Li et al. (2013). The identical genetic context of antibiotic resistance genes on IncHI2 plasmids was also detected in *S.* Indiana in China (Lai et al., 2013) as well as *S.* Typhimurium in Europe (Campos et al., 2015), suggesting a similar evolutionary origin and highlighting the potentially global spread of IncHI2 plasmids among *Salmonella.* Furthermore, a similar genetic context of *aac(6′)-Ib-cr* with *bla*_OXA-1_ was also identified on an IncR plasmid of a *K. oxytoca* strain in Spain (Ruiz et al., 2011), and an IncN plasmid of an *E. coli* isolate in Hong Kong (Ho et al., 2013). The similar resistance gene module infers that the transfer of resistance-associated module was driven by mobile genetic elements like integrons, insertion sequences or transposons between different plasmid replicons, and was probably mediated by IS*26* in terms of the above cases (Miriagou et al., 2005; Li et al., 2013; He et al., 2015). Thus, further active surveillance is needed to minimize the spread of particular plasmids such as IncHI2 group to control the dissemination of antibiotic resistance. As mobile genetic elements play a major role in the acquisition and dissemination of antibiotic resistance genes, further study may be required to determine the distinct genetic environment of antibiotic resistance genes on plasmids.

### Analysis of pS10584

The MDR *Salmonella enterica* serovar Indiana isolate SJTUF 10584 identified in this study was chosen as the plasmid donor. This isolate contains IncHI2 and IncI1 plasmids simultaneously, and these two types of plasmids were easily eliminated after one trial of curing. The plasmid designated as pS10584 was acquired through conjugation experiments recovered from ampicillin and rifampin resistance selection. Replicon typing showed that the only transferred plasmid pS10584 belonged to the IncI1 group and PCR analysis confirmed that it was positive for *bla*_CMY -2_ gene but not for *bla*_OXA-1_ or *aac(6′)-Ib-cr* genes. All of the transconjugants shared the same antibiotic resistance pattern of AMP-SAM-CZO-CAZ-CRO.

**FIGURE 3:**
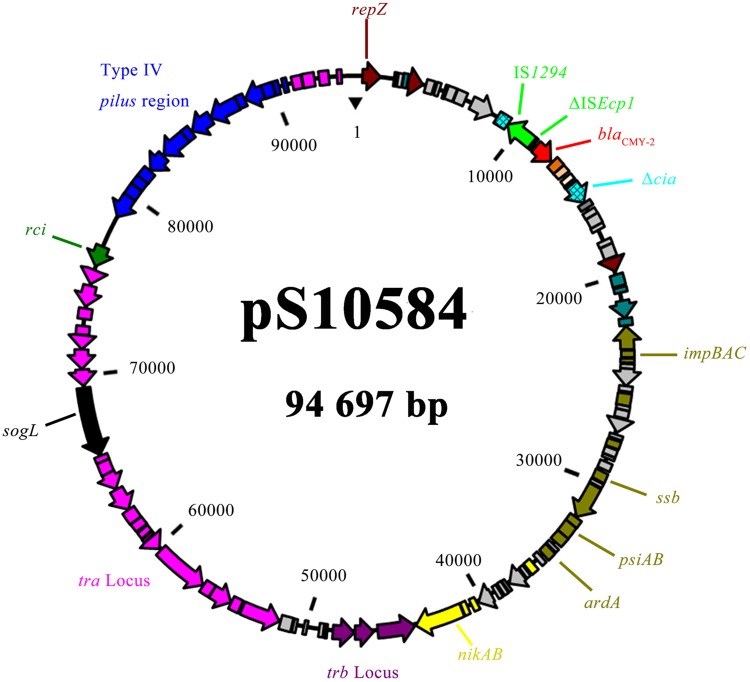
**An overview of the *bla*_CMY -2_-carrying plasmid pS10584.** Genes are color-coded as follows: dark brown, replication-associated genes; green, insertion sequences; red, antibiotic resistance gene; blackish green, partition- or stability-associated genes; army green, transfer-leading region associated genes; pink, *tra* locus; blue, type IV pilus region; purple, *trb* locus; and gray, hypothetical protein.

Complete DNA sequence of pS10584 revealed that it was a circular molecule of 94,697-bp with an average G+C content of 50.0% and 110 putative CDSs. In general, pS10584 possessed a plasmid scaffold typical for IncI1 plasmids, with important IncI1-associated genetic modules including *traABCD* regulatory cluster, *traLMNOPQRSTUVWXY* transfer region, *nikB-trbABC* region, *ssb-psiAB* region, and type IV thin pilus formation region *pilIJKLMNOPQRSTUV* (**Figure 3**). Besides the approximate 89,866-bp “core genome” region encoding characteristic IncI1 plasmid replication, transfer, maintenance and stability functions, a 4,831-bp accessory module encoding the antibiotic resistance gene as well as flanking insertion sequences was also discovered. BLASTN comparison revealed that the overall genetic organization of pS10584 was very similar to that of another fully sequenced IncI1 plasmid pJIE512b (GenBank accession no. HG970648; Tagg et al., 2014) with high sequence identity (>99% at nucleotide level) except an inversion within the *pilV* shuﬄon and a 2,307-bp deletion (nucleotide 6,685^th^ to 8,991^nd^ of pS10584, containing a truncated colicin gene *cia* and an intact putative DNA-binding transcriptional regulator gene *yagA*) in pJIE5112b.

**FIGURE 4:**
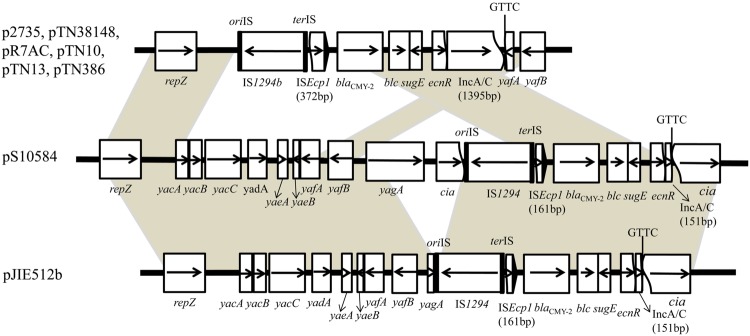
**Genetic context of the *bla*_CMY -2_ gene in plasmid pS10584 of *Salmonella* Indiana isolate SJTUF 10584 and structure comparison with plasmids p2735 from *Klebsiella oxytoca* and pTN38148, pTN10, pTN13, pTN386, pR7AC, pJIE512b from *Escherichia coli*.** Regions of >99% homology are marked by gray shading.

In pS10584, the 4,831-bp antibiotic resistance-associated module comprised 161-bp of the right end of IS*Ecp1* truncated by IS*1294* in the reverse orientation and a 2,823-bp region originating from the *Citrobacter freundii* chromosome including a *bla*_CMY -2_ gene, with *blc* (an outer membrane lipoprotein, lipocalin), *sugE* (a small MDR transporter), and Δ*ecnR* (LuxR family transcriptional regulator) genes immediately downstream, followed by a 159-bp fragment of IncA/C ending in GTTC which matched the last 4-bp of *ter*IS of IS*1294* (**Figure 4**). In addition, the 4831-bp *bla*_CMY -2_ segment inserted within the *cia* gene at the 698-bp from the 5′ end without causing adjacent deletion, followed by the remaining 1,184-bp segment of the *cia* gene.

A similar genetic context surrounding the *bla*_CMY -2_ gene was identified in an IncI1 plasmid p2735 (KP017243; Yassine et al., 2014) isolated from *Klebsiella pneumoniae* as well as six IncI1 plasmids isolated from *E. coli* including two completely sequenced plasmids pJIE512b (HG970648; Tagg et al., 2014), and pR7AC (KF434766; Minoia et al., 2012), and four partially sequenced plasmids pTN38148 (FM246883), pTN10, pTN13, and pTN386 (Verdet et al., 2009) (**Figure 4**). Compared to pS10584, the *bla*_CMY -2_ insertion was within the *yagA* gene 16-bp from the 5′ end, removing the rest of the *yagA* gene, as well as a large part of the *cia* gene, leaving the same part of the *cia* gene in the 3′ end followed by the same 159-bp IncA/C backbone ending in GTTC in pJIE512b. Whereas, the flanking fragments of *bla*_CMY -2_ gene in other six IncI1 plasmids (pTN10, pTN13, pTN386, pTN38148, p2735, and pR7AC) were much more different from that in pS10584, including an IS*1294*-like element designated as IS*1294b* (Yassine et al., 2014), followed by a 372-bp truncated IS*Ecp1*, the identical 2,823-bp region of the *C. freundii* chromosome that contained *bla*_CMY -2_ and the additional 1395-bp IncA/C backbone also ending in GTTC (**Figure 4**). This *bla*_CMY -2_ insertion was downstream of the *repZ* gene, removing a larger adjacent part and truncating the 3′ end of *yafA* followed by *yafB*.

Although *S.* Indiana frequently exhibited MDR phenotypes, very little research has been done to clarify the molecular mechanisms conferring its MDR or the transferability of the resistance properties (Lai et al., 2013). Among *ampC*-like genes, the *bla*_CMY -2_ gene which probably originated from *C. freundii*, is the most prevalent gene worldwide conferring resistance to clinically important *β*-lactam-based compounds particularly the third-generation cephalosporins. (Verdet et al., 2009; Call et al., 2010; Karczmarczyk et al., 2014; Tagg et al., 2014). In this study, two *bla*_CMY -2_-positive isolates both showed resistance phenotypes against the third-generation cephalosporins (CRO, CAZ, and CZO) while the other one was susceptible to them, probably due to the low-level expression or silence of this gene. In previous research, it was observed that resistance to a second generation cephalosporin, cefoxitin in *Salmonella* isolates harboring *bla*_CMY -2_ (Giles et al., 2004; Martin et al., 2012; Noda et al., 2015). However, none of the three *bla*_CMY -2_-positive isolates was resistant to cefotetan, which is a second-generation cephalosporin like cefoxitin. This was also found in a previous study (Noda et al., 2015). This may also be attributed to the low-level expression or silence of *bla*_CMY -2_ gene.

Among various plasmid types associated with the carriage of the *bla*_CMY -2_ gene, IncI1 and IncA/C plasmids are often reported as the predominant carriers (Mata et al., 2012; Bortolaia et al., 2014). Additional research discovered that *bla*_CMY -2_ carrying IncA/C plasmids always conferred additional antimicrobial resistance phenotypes such as chloramphenicol and sulfisoxazole, whereas in this study, *bla*_CMY -2_ carrying IncI1 plasmids only conferred a *bla*_CMY -2_-associated phenotype, which was also observed in Folster et al. (2011). The *bla*_CMY -2_ genetic context on the IncI1 plasmid identified in this study was IS*1294*-*Δ*IS*Ecp1*-*bla*_CMY -2_-*blc*-*sugE*-*ΔecnR* followed by 159-bp IncA/C backbone, which was identical to that of pJIE512b from an *E. coli* isolate (Tagg et al., 2014). The genetic arrangement IS*Ecp1*-*bla*_CMY -2_-*blc*-*sugE*-*ecnR* was conserved on IncA/C plasmids (Verdet et al., 2009; Ye et al., 2016). IS*Ecp1* was presumed to be responsible for the mobilization of *bla*_CMY -2_ (Verdet et al., 2009) or *bla*_CTX-M-2_ (Lartigue et al., 2006) as well as its adjacent region from the bacterial chromosome to the IncA/C plasmids by a one-ended mobilization mechanism. On the basis of sequence alignments, it was suggested that mobilization of *bla*_CMY -2_ from the IncA/C plasmid to the IncI1 replicon occurred through IS*1294*-mediated transposition activity (Tagg et al., 2014). The *bla*_CMY -2_ insertion region of pS10584 started in CTTG and ended in GTTC, both of which matched the last 4-bp of ori*IS* and ter*IS* of IS*1294* (Tavakoli et al., 2000; Yassine et al., 2014), implying that I*S1294* inserted into IS*Ecp1* in an IncA/C plasmid and subsequently mobilized the adjacent region, including the *bla*_CMY -2_ segment plus a 159-bp IncA/C backbone (Tagg et al., 2014). A similar *bla*_CMY -2_ context was also observed in six reported IncI1 plasmids p2735 (Yassine et al., 2014), pR7AC (Minoia et al., 2012), pTN38148, TN10, TN13, and TN386 (Verdet et al., 2009); however, in these plasmids the *bla*_CMY -2_ insertion segment varied in the length of IncA/C backbone and the position that IS*Ecp1* interrupted by an IS*1294*-like element designated as IS*1294b*. And the integration of the *bla*_CMY -2_ gene by IS*1294b* between IncA/C and IncI1 replicons was experimentally confirmed by Yassine et al. (2014). By comparing the integration sites of *bla*_CMY -2_ insertion segments on these reported IncI1 plasmids, we inferred that the downstream of *repZ* gene could represent a “hot spot” for the integration of IS*1294b* element in the IncI1 plasmid scaffold but determination of the “hot spot” for IS*1294* may require further study.

Besides the resistance determinants IncI1 plasmids encode, additional factors may be responsible for their prevalent diffusion. The shuﬄon multiple inversion system on IncI1 plasmids involves four segments separated by seven inverted repeats and a shuﬄon-specific recombinase (Rci) which acts to catalyze recombination between these repeats (Sampei et al., 2010). This shuﬄon system is responsible for generating variation in the C terminus of the *pilV* tip adhesins of the pili, leading to the specificity of recipient cells in liquid mating (Tagg et al., 2014). The resulting type IV pilus is also a known virulence factor, contributing to adhesion and invasion of Shiga toxin-producing *E. coli* (Kim and Komano, 1997), which accounts for the prevalent existence of IncI1 plasmids in pathogenic *E. coli* (Johnson et al., 2007). Thus, the association of epidemic ability as well as resistance determinants may favor the wide dissemination of plasmids belonging to the IncI1 group (Carattoli, 2009; Folster et al., 2010).

## Conclusion

We screened and characterized antibiotic resistance plasmids from clinical and food *Salmonella* isolates in this study and discovered that IncHI2 was the major plasmid lineage contributing to the dissemination of antibiotic resistance in *Salmonella*. We also reported the complete sequence of an IncI1 plasmid harboring an antibiotic resistance-encoding region with the genetic arrangement of IS*1294*-*Δ*IS*Ecp1*-*bla*_CMY -2_-*blc*-*sugE*-*ΔecnR*, interrupting the *cia* gene from a MDR *S.* Indiana isolate. To our knowledge, this is the first report of this *bla*_CMY -2_ context on the IncI1 plasmid in *Salmonella*. These results revealed that plasmids represent a potential threat for the dissemination of antibiotic resistance since they carry relevant resistance determinants and complete transfer-associated genes. Thus, in addition to phenotypic monitoring of antimicrobial resistance in *Salmonella*, further active surveillance is needed to minimize the spread of particular plasmids such as those belonging to the IncHI2 group. In addition, investigations of mobile genetic elements that are located on plasmids, such as insertion sequences, integrons, and transposons, may aid in understanding the source and spread of antibiotic resistance.

## Author Contributions

WC completed the antimicrobial susceptibility test, PMQR and β-lactamase gene detection, plasmid replicon typing, plasmid curing, plasmid sequencing, and prepare the manuscript; TF completed the isolate collection, antimicrobial susceptibility test, PMQR and β-lactamase gene detection, plasmid replicon typing, and plasmid curing; XZ helped to finish the isolate collection and antimicrobial susceptibility test; DZ helped to finish the plasmid sequencing and analysis; XS helped to design the project and give some valuable suggestions; and CS designed the project, completed the data analysis, and prepared the manuscript.

## Conflict of Interest Statement

The authors declare that the research was conducted in the absence of any commercial or financial relationships that could be construed as a potential conflict of interest.

## Footnotes

1 http://blast.ncbi.nlm.nih.gov/Blast.cgi

2 https://www-is.biotoul.fr//
