# Supplementary material for: IncHI2 Plasmids Are Predominant in Antibiotic-Resistant Salmonella Isolates
Source: Front Microbiol. 2016 Sep 30;7:1566. doi: 10.3389/fmicb.2016.01566 (PMC5043248; doi:10.3389/fmicb.2016.01566)
Supplement: Supplementary file 1 [file Table_1.docx]

Supplementary Material

IncHI2 Plasmids Are Predominant in Antibiotic Resistant *Salmonella* Isolates

Wenyao Chen^†,^ Tingzi Fang^†^, Xiujuan Zhou, Daofeng Zhang, Xianming Shi, Chunlei Shi

*** Correspondence:** Chunlei Shi: [clshi@sjtu.edu.cn](mailto:clshi@sjtu.edu.cn)

**^†^** Wenyao Chen and Tingzi Fang have contributed equally to this work.

**Table S1.** Non-susceptible *Salmonella* isolates used in this study (*n*=78).

| **Isolate** | **Serovar** | **Year** | **Region** | **Sample type** | **Isolate** | **Serovar** | **Year** | **Region** | **Sample type** |
| --- | --- | --- | --- | --- | --- | --- | --- | --- | --- |
| SJTUF10023 | Typhimurium | 2006 | Shanghai | Feces | SJTUF10207 | Indiana | 2007 | Shanghai | Feces |
| SJTUF10057 | Typhimurium | 2006 | Shanghai | Feces | SJTUF10476 | Indiana | 2007 | Shanghai | Chicken |
| SJTUF10112 | Typhimurium | 2006 | Shanghai | Feces | SJTUF10566 | Indiana | 2006 | Shanghai | Chicken |
| SJTUF10155 | Typhimurium | 2006 | Shanghai | Feces | SJTUF10584 | Indiana | 2006 | Shanghai | Chicken |
| SJTUF10157 | Typhimurium | 2006 | Shanghai | Feces | SJTUF10585 | Indiana | 2006 | Shanghai | Chicken |
| SJTUF10169 | Typhimurium | 2006 | Shanghai | Feces | SJTUF10702 | Indiana | 2006 | Wuhan | Chicken |
| SJTUF10211 | Typhimurium | 2007 | Shanghai | Feces | SJTUF10713 | Heidelberg | 2006 | Wuhan | Chicken |
| SJTUF10231 | Typhimurium | 2007 | Shanghai | Feces | SJTUF10740 | Heidelberg | 2006 | Wuhan | Chicken |
| SJTUF10233 | Typhimurium | 2007 | Shanghai | Feces | SJTUF10772 | Heidelberg | 2006 | Wuhan | Feces |
| SJTUF10236 | Typhimurium | 2007 | Shanghai | Feces | SJTUF10456 | Derby | 2007 | Shanghai | Pork |
| SJTUF10250 | Typhimurium | 2007 | Shanghai | Feces | SJTUF10469 | Derby | 2007 | Shanghai | Pork |
| SJTUF10327 | Typhimurium | 2007 | Shanghai | Feces | SJTUF10475 | Derby | 2007 | Shanghai | Pork |
| SJTUF10328 | Typhimurium | 2007 | Shanghai | Feces | SJTUF10560 | Derby | 2006 | Shanghai | Pork |
| SJTUF10329 | Typhimurium | 2007 | Shanghai | Feces | SJTUF10589 | Derby | 2006 | Shanghai | Pork |
| SJTUF10330 | Typhimurium | 2007 | Shanghai | Feces | SJTUF10754 | Derby | 2006 | Wuhan | Feces |
| SJTUF10479 | Typhimurium | 2007 | Shanghai | Chicken | SJTUF10054 | Anatum | 2006 | Shanghai | Feces |
| SJTUF10484 | Typhimurium | 2007 | Shanghai | Clam | SJTUF10482 | Anatum | 2007 | Shanghai | Pork |
| SJTUF10565 | Typhimurium | 2006 | Shanghai | Chicken | SJTUF10580 | Anatum | 2006 | Shanghai | Pork |
| SJTUF10567 | Typhimurium | 2006 | Shanghai | Pork | SJTUF10762 | Anatum | 2006 | Wuhan | Feces |
| SJTUF10568 | Typhimurium | 2006 | Shanghai | Pork | SJTUF10230 | Agona | 2007 | Shanghai | Feces |
| SJTUF10570 | Typhimurium | 2006 | Shanghai | Pork | SJTUF10247 | Agona | 2007 | Shanghai | Feces |
| SJTUF10577 | Typhimurium | 2006 | Shanghai | Saury | SJTUF10249 | Agona | 2007 | Shanghai | Feces |
| SJTUF10578 | Typhimurium | 2006 | Shanghai | Pork | SJTUF10725 | Agona | 2007 | Wuhan | Feces |
| SJTUF10586 | Typhimurium | 2006 | Shanghai | Chicken | SJTUF10711 | Manhattan | 2007 | Wuhan | Beef |
| SJTUF10694 | Typhimurium | 2006 | Wuhan | Beef | SJTUF10750 | Manhattan | 2007 | Wuhan | Pork |
| SJTUF10024 | Enteritidis | 2006 | Shanghai | Feces | SJTUF10758 | Manhattan | 2007 | Wuhan | Pork |
| SJTUF10029 | Enteritidis | 2006 | Shanghai | Feces | SJTUF10213 | Thompson | 2007 | Shanghai | Feces |
| SJTUF10229 | Enteritidis | 2007 | Shanghai | Feces | SJTUF10703 | Thompson | 2007 | Shanghai | Shrimp |
| SJTUF10331 | Enteritidis | 2007 | Shanghai | Feces | SJTUF10051 | Aberdeen | 2006 | Shanghai | Feces |
| SJTUF10459 | Enteritidis | 2007 | Shanghai | Carrot | SJTUF10701 | Infantis | 2007 | Wuhan | Pork |
| SJTUF10462 | Enteritidis | 2007 | Shanghai | Chicken | SJTUF10782 | Kentucky | 2007 | Wuhan | Pork |
| SJTUF10491 | Enteritidis | 2007 | Shanghai | Chicken | SJTUF10768 | Litchfield | 2007 | Wuhan | Feces |
| SJTUF10571 | Enteritidis | 2006 | Shanghai | Chicken | SJTUF10483 | Mbandaka | 2007 | Shanghai | Pork |
| SJTUF10587 | Enteritidis | 2006 | Shanghai | Duck | SJTUF10216 | Montevideo | 2007 | Shanghai | Feces |
| SJTUF10717 | Enteritidis | 2006 | Wuhan | Feces | SJTUF10721 | Paratyphi.A | 2007 | Wuhan | Feces |
| SJTUF10718 | Enteritidis | 2006 | Wuhan | Feces | SJTUF10485 | Paratyphi.B | 2007 | Shanghai | Pork |
| SJTUF10720 | Enteritidis | 2006 | Wuhan | Feces | SJTUF10573 | Stanley | 2006 | Shanghai | Razor clam |
| SJTUF10119 | Braenderup | 2006 | Shanghai | Feces | SJTUF10700 | Virchow | 2007 | Wuhan | Orange juice |
| SJTUF10334 | Braenderup | 2007 | Shanghai | Feces | SJTUF10705 | Typhi | 2007 | Wuhan | Feces |
