# Supplementary material for: IncHI2 Plasmids Are Predominant in Antibiotic-Resistant Salmonella Isolates
Source: Front Microbiol. 2016 Sep 30;7:1566. doi: 10.3389/fmicb.2016.01566 (PMC5043248; doi:10.3389/fmicb.2016.01566)
Supplement: Supplementary file 2 [file Table_2.DOCX]

Supplementary Material

IncHI2 Plasmids Are Predominant in Antibiotic Resistant *Salmonella* Isolates

Wenyao Chen^†,^ Tingzi Fang^†^, Xiujuan Zhou, Daofeng Zhang, Xianming Shi, Chunlei Shi

*** Correspondence:** Chunlei Shi: [clshi@sjtu.edu.cn](mailto:clshi@sjtu.edu.cn)

**^†^** Wenyao Chen and Tingzi Fang have contributed equally to this work.

**Table S2**. Two different primer pairs and an additional primer pair used in *Salmonella* serovar detection

| **Target gene**  **(serogroup)** | **Nucleotide sequence (5’→3’)** | **Designation** | **Amplicon size (bp)** | **Reference** |
| --- | --- | --- | --- | --- |
| *wzx* (O:7 [C1]) | AGTGGTAGGAGGGGATAGGT  TGTTCGATAGAGTAAATGATGCT | Multiplex PCR 2 | 438 | This study |
| *wzx* (O:8 [C2-C3]) | ACTAAAACGGAGGGGCTATGA  TACCCACCATCCACGAACTAA | Multiplex PCR 2 | 173 | Thist study |
| *wzx* (O:11 [F]) | GTCTTCCCCTGGCATTGTTG  CTCATAATGCCCATGCGAGAC | Multiplex PCR 2 | 307 | This study |
