# Supplementary material for: IncHI2 Plasmids Are Predominant in Antibiotic-Resistant Salmonella Isolates
Source: Front Microbiol. 2016 Sep 30;7:1566. doi: 10.3389/fmicb.2016.01566 (PMC5043248; doi:10.3389/fmicb.2016.01566)
Supplement: Supplementary file 3 [file Table_3.DOCX]

Supplementary Material

IncHI2 Plasmids Are Predominant in Antibiotic Resistant *Salmonella* Isolates

Wenyao Chen^†,^ Tingzi Fang^†^, Xiujuan Zhou, Daofeng Zhang, Xianming Shi, Chunlei Shi

*** Correspondence:** Chunlei Shi: [clshi@sjtu.edu.cn](mailto:clshi@sjtu.edu.cn)

**^†^** Wenyao Chen and Tingzi Fang have contributed equally to this work.

**Table S3.** Oligonucleotides primers for the detection of plasmid-mediated quinolone resistance (PMQR) genes and *β*-lactamase genes

| **Target gene** | **Nucleotide sequence (5’→3’)** | **Reference** |
| --- | --- | --- |
| *bla*_CMY-2_ | TGGCCGTTGCCGTTATCTAC  CCCGTTTTATGCACCCATGA | [Weill et al. (2006)](#_ENREF_8) |
| *bla*_OXA-1_ | ATGAAAAACACAATACATATC  AATTTAGTGTGTTTAGAATGG | [Casin et al. (1999)](#_ENREF_1) |
| *bla*_TEM-1_ | ATAAAATTCTTGAAGACGAAA  GACAGTTACCAATGCTTAATC | [Mabilat and Goussard (1993)](#_ENREF_5) |
| *bla*_PSE-1_ | CGCTTCCCGTTAACAAGTAC  CTGGTTCATTTCAGATAGCG | [Sandvang et al. (1998)](#_ENREF_6) |
| *qnrA* | AGAGGATTTCTCACGCCAGG  TGCCAGGCACAGATCTTGAC | [Cattoir et al. (2007)](#_ENREF_2) |
| *qnrB* | GGMATHGAAATTCGCCACTG  TTTGCYGYYCGCCAGTCGAA | [Cattoir et al. (2007)](#_ENREF_2) |
| *qnrS* | GCAAGTTCATTGAACAGGGT  TCTAAACCGTCGAGTTCGGCG | [Cattoir et al. (2007)](#_ENREF_2) |
| *qnrC* | GGGTTGTACATTTATTGAATC  TCCACTTTACGAGGTTCT | [Wang et al. (2009)](#_ENREF_7) |
| *qnrD* | GGGGATATCTTAAGGTTGTTCAAATTAATGTAC  CCCGTCGACTTTGATTAGTACCACATTGG | [Cavaco et al. (2009)](#_ENREF_3) |
| *aac(6’)-Ib-cr* | TTGCGATGCTCTATGAGTGGCTA  CTCGAATGCCTGGCGTGTTT | [Cavaco et al. (2008)](#_ENREF_4) |

Casin, I., Breuil, J., Brisabois, A., Moury, F., Grimont, F., and Collatz, E. (1999). Multidrug-resistant human and animal Salmonella typhimuvium isolates in France belong predominantly to a DT104 Clone with the chromosome-and integron-encoded β-Lactamase PSE-1. *Journal of Infectious Diseases* 179(5)**,** 1173-1182.

Cattoir, V., Poirel, L., Rotimi, V., Soussy, C.-J., and Nordmann, P. (2007). Multiplex PCR for detection of plasmid-mediated quinolone resistance qnr genes in ESBL-producing enterobacterial isolates. *Journal of Antimicrobial Chemotherapy* 60(2)**,** 394-397.

Cavaco, L., Hasman, H., Xia, S., and Aarestrup, F.M. (2009). qnrD, a novel gene conferring transferable quinolone resistance in Salmonella enterica serovar Kentucky and Bovismorbificans strains of human origin. *Antimicrobial agents and chemotherapy* 53(2)**,** 603-608.

Cavaco, L.M., Frimodt-Møller, N., Hasman, H., Guardabassi, L., Nielsen, L., and Aarestrup, F.M. (2008). Prevalence of quinolone resistance mechanisms and associations to minimum inhibitory concentrations in quinolone-resistant Escherichia coli isolated from humans and swine in Denmark. *Microbial Drug Resistance* 14(2)**,** 163-169.

Mabilat, C., and Goussard, S. (1993). PCR detection and identification of genes for extended-spectrum β-lactamases. *Diagnostic molecular microbiology: principles and applications. American Society for Microbiology, Washington, DC***,** 553-559.

Sandvang, D., Aarestrup, F.M., and Jensen, L.B. (1998). Characterisation of integrons and antibiotic resistance genes in Danish multiresistant Salmonella enterica Typhimurium DT104. *FEMS microbiology letters* 160(1)**,** 37-41.

Wang, M., Guo, Q., Xu, X., Wang, X., Ye, X., Wu, S., et al. (2009). New plasmid-mediated quinolone resistance gene, qnrC, found in a clinical isolate of Proteus mirabilis. *Antimicrobial agents and chemotherapy* 53(5)**,** 1892-1897.

Weill, F.-X., Guesnier, F., Guibert, V., Timinouni, M., Demartin, M., Polomack, L., et al. (2006). Multidrug resistance in Salmonella enterica serotype Typhimurium from humans in France (1993 to 2003). *Journal of clinical microbiology* 44(3)**,** 700-708.
